# The Transcription Factor YY1 Is a Substrate for Polo-Like Kinase 1 at the G2/M Transition of the Cell Cycle

**DOI:** 10.1371/journal.pone.0015928

**Published:** 2011-01-06

**Authors:** Raed Rizkallah, Karen E. Alexander, Ari Kassardjian, Bernhard Lüscher, Myra M. Hurt

**Affiliations:** 1 Department of Biomedical Sciences, Florida State University, Tallahassee, Florida, United States of America; 2 Institute of Molecular Biophysics, Florida State University, Tallahassee, Florida, United States of America; 3 Institut für Biochemie und Molekularbiologie, Rheinisch-Westfälische Technische Hochschule Aachen University, Aachen, Germany; University of Minnesota, United States of America

## Abstract

Yin-Yang 1 (YY1) is an essential multifunctional zinc-finger protein. It has been shown over the past two decades to be a critical regulator of a vast array of biological processes, including development, cell proliferation and differentiation, DNA repair, and apoptosis. YY1 exerts its functions primarily as a transcription factor that can activate or repress gene expression, dependent on its spatial and temporal context. YY1 regulates a large number of genes involved in cell cycle transitions, many of which are oncogenes and tumor-suppressor genes. YY1 itself has been classified as an oncogene and was found to be upregulated in many cancer types. Unfortunately, our knowledge of what regulates YY1 is very minimal. Although YY1 has been shown to be a phosphoprotein, no kinase has ever been identified for the phosphorylation of YY1. Polo-like kinase 1 (Plk1) has emerged in the past few years as a major cell cycle regulator, particularly for cell division. Plk1 has been shown to play important roles in the G/M transition into mitosis and for the proper execution of cytokinesis, processes that YY1 has been shown to regulate also. Here, we present evidence that Plk1 directly phosphorylates YY1 *in vitro* and *in vivo* at threonine 39 in the activation domain. We show that this phosphorylation is cell cycle regulated and peaks at G2/M. This is the first report identifying a kinase for which YY1 is a substrate.

## Introduction

YY1 is a ubiquitously expressed multifunctional transcription factor that has been shown to be involved in the regulation of a large number of genes that are critical for basic biological processes of cell growth, development, differentiation, cell cycle and even programmed cell death (apoptosis) (reviewed in [1], [2]). YY1 is an essential protein; its complete ablation was shown to cause lethality in mice at day seven of embryogenesis and disruption of one allele causes severe developmental defects [3].

The structural and functional domains of the YY1 protein have been well characterized. YY1 is a sequence-specific DNA binding C_2_H_2_ zinc finger protein that contains both a transactivation domain and a repression domain [4], [5]. The role of YY1 in cellular proliferation has been proposed since its discovery [6]. This was further supported by identification of several cell cycle regulators that are modulated by YY1, like c-Myc [7], [8], [9], RB [10], [11], p53 [12], [13], [14], [15], and many others. In addition, knockdown of YY1 was shown to reduce cell proliferation and cause an accumulation of multinucleated cells with a variety of nuclear abnormalities [16]. This is possibly due to a role for YY1 in the regulation of cytokinesis. This role could be direct or indirect. In the analysis of the effects of YY1 knockdown on gene expression, a cluster of genes normally upregulated at G2/M was found to be down-regulated [16].

The involvement of YY1 in cell proliferation and regulation of oncogenes and tumor-suppressor genes has led several groups to investigate the role of YY1 in tumor development (reviewed in [2], [17], [18], [19]. For example, elevated YY1 levels were detected in many tumor types including prostate cancer [20], [21], ovarian cancer [19], colon cancer [22], breast cancer [22], cervical cancer [23], osteosarcoma [24], acute myeloid leukemia [25], [26], Hodgkin's lymphoma [19], [27], non-Hodgkin's lymphoma [28], and follicular lymphoma [29]. In addition, higher YY1 transcript and protein levels were associated with malignant transformation in cervical cancer, in the presence of a Human Papilloma Virus (HPV) infection [30].

Although a substantial amount of information has been compiled over the past decade about target genes regulated by YY1, much less evidence has been gathered to provide a model for its mode of action and, more importantly, its regulation. The expression and protein levels of YY1 remain constant across the different phases of the cell cycle [31], [32], [33]. This leads to the possibility that YY1 is regulated by post-translational modification, phosphorylation in particular, to play specific roles at specific time points in the cell cycle. We have previously reported that phosphorylation of YY1 in the DNA binding domain during mitosis abolishes its DNA binding activity [31]. Also, several large scale proteomics studies have mapped phosphorylation sites on YY1, including serines 118, 184, 247, threonines 348 and 378 [34], [35], [36], [37] but no particular kinase has ever been identified.

Polo-like kinase one (Plk1) is a serine/threonine kinase, initially identified in *Drosophila* as Polo, and shown to play pivotal roles in proper spindle pole formation [38]. In mammalian cells, Plk1 is a critical regulator of several important cell cycle events [39]. Primarily, it is part of the intricate network orchestrating the accurate and timely execution of mitosis and cytokinesis [40], [41], [42]. Plk1 has received significant interest in the past few years due to its role in cell proliferation and its upregulation in many types of cancer, leading to its targeting in cancer therapies [43], [44], [45], [46], [47], [48]. Clinical trials are already underway [43]. Many groups have focused on elucidating the various roles played by Plk1 in cell division by identifying an array of substrates, including enzymes, spindle checkpoint proteins, and structural proteins [49], [50], [51], [52], [53], [54], [55], [56], [57], [58], [59], [60]. Recently, a study has found the first direct link between Plk1 and transcriptional regulation at G2/M, by identifying Forkhead Box M1 (FoxM1) transcription factor as a physiological substrate for Plk1 [61]. FoxM1 is an important activator of a cluster of genes needed for progression into mitosis. Plk1 phosphorylation of FoxM1 was shown to provide a positive feedback loop for mitotic entry [61].

Here, we report the first identification of a kinase that phosphorylates the transcription factor YY1. We identify a novel phosphorylation site in the activation domain of YY1 and show that this phosphorylation is cell cycle regulated, peaking at G2/M. We show that Plk1 is the kinase that phosphorylates YY1 at this site, revealing an additional link between Plk1 and the transcriptional machinery at the entry of mitosis.

## Results

### YY1 is a direct substrate for Plk1 *in vitro*

Due to the critical role that the transcription factor YY1 plays in the life of mammalian cells, and its link to cancer, there is an urgent need for a better understanding of its regulation. Although YY1 has been proposed [62] and shown to be a phosphoprotein [31], there is no evidence in the scientific literature of a phosphorylation signaling pathway which directly regulates YY1. More specifically, YY1 has never been shown to be the direct substrate of any specific kinase.

**Figure 1:**
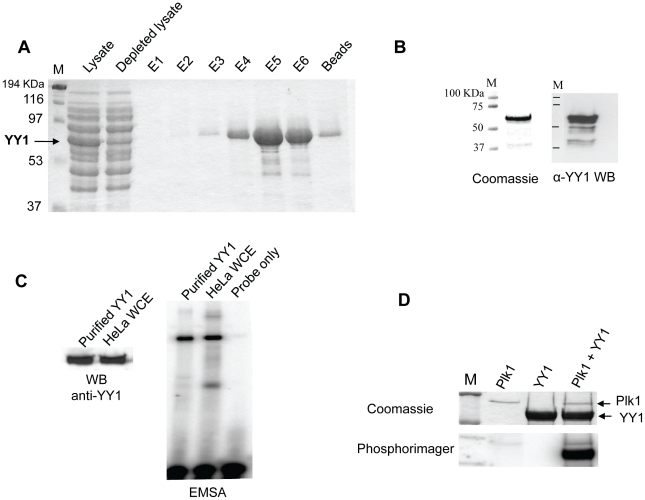
Plk1 phosphorylates YY1 *in vitro*. (**A**) Coomassie blue staining of SDS-PAGE gel showing the purification of bacterially expressed non-tagged YY1 under denaturing conditions. An arrow indicates the position of overexpressed YY1. Lysates: sample from total bacterial lysates; Depleted lysates: sample from bacterial lysates after passing through the Ni-NTA column. E1, 2, and 3 are samples from elutions at pH 5.9; E4, 5, and 6 are samples from elutions at pH 4.5; the last lane shows a sample of the Ni-NTA beads after the last elution step. (**B**) Coomassie blue staining (left panel) and Western blot analysis (right panel) of purified YY1 after renaturation, and separation by SDS-PAGE. In the Western blot, YY1 was probed with anti-YY1 (H-10) antibody. (**C**) EMSA testing the DNA binding activity of purified and renatured YY1 side by side with HeLa whole cell extract. A 22 bp oligonucleotide encompassing the YY1 binding site in histone H3.2 coding region was used as the radioactively labeled probe. The Western blot shows equal amounts of purified YY1 and YY1 protein in HeLa WCEs. (**D**) Coomassie blue staining and phosphorimager exposure of SDS-PAGE gel after radioactive *in vitro* kinase assay, using purified Plk1 and YY1. The gel shows three reactions: Plk1 only, YY1 only, and Plk1 plus YY1; as indicated.

To explore these signaling pathways and screen various upstream kinases, we developed a novel method for the purification of bacterially expressed YY1, without the need for any tagging system. The N-terminal domain of YY1 contains a stretch of 11 histidine residues that can potentially fulfill the role of a His-tag. Initial attempts to purify YY1, using this natural His tag, under native conditions, were not successful. This was most likely due to shielding of the histidine stretch in the three dimensional folding of the protein. However, when the purification was performed under denaturing conditions, high yields of pure YY1 were readily produced (Fig. 1A). After optimizing renaturation conditions, as described in the Material and Methods section, we were able to obtain pure YY1 protein (Fig. 1 B). YY1 is a transcription factor, and its DNA binding activity has been well characterized. Therefore, we tested its proper functional folding using an *in vitro* DNA binding assay. Renatured YY1 was able to bind its DNA consensus site found in the coding region of Histone H3.2, in an electrophoretic-mobility shift assay (EMSA), as previously described (Fig. 1C) [63]. In addition, to provide evidence that most of the renatured YY1 was functional, we used comparable amounts of purified YY1 to those found in HeLa whole cell extracts. As shown in Figure 1C, equal amounts of the YY1 protein from the two sources generated comparable shifts in the EMSA assay. Purified YY1 was also able to bind several previously identified consensus binding sites from different biological contexts [6], [64] in EMSA assays (data not shown).

To search for kinases that could phosphorylate YY1, we used the GPS 2.0 (Group-based Prediction System) software **(** http://gps.biocuckoo.org **)** [65]. Several kinases were predicted to phosphorylate YY1 at distinct residues. Since we were particularly interested in the role and regulation of YY1 in the context of cell proliferation, we narrowed our search to the kinases that are involved in cell cycle regulation. The main kinase of this category which had several consensus sites in YY1 was Plk1.

To test if YY1 is indeed a good substrate for Plk1, we performed a radioactive *in vitro* kinase assay using purified YY1 and Plk1, expressed and purified from insect cells (SignalChem). As shown in Figure 1D, Plk1 was able to phosphorylate YY1 *in vitro*. YY1 alone did not show any kinase or autophosphorylation activity (Fig. 1D), as previously reported [1], [31].

### Plk1 phosphorylates YY1 at threonine 39 in the N-terminal activation domain

Several sites on YY1 were predicted to be potential phosphorylation sites for Plk1, and resembled previously reported Plk1 phosphorylation sites on other proteins [59], [66]. To map the exact residue(s) that Plk1 phosphorylates on YY1, we followed a two-step approach.

**Figure 2:**
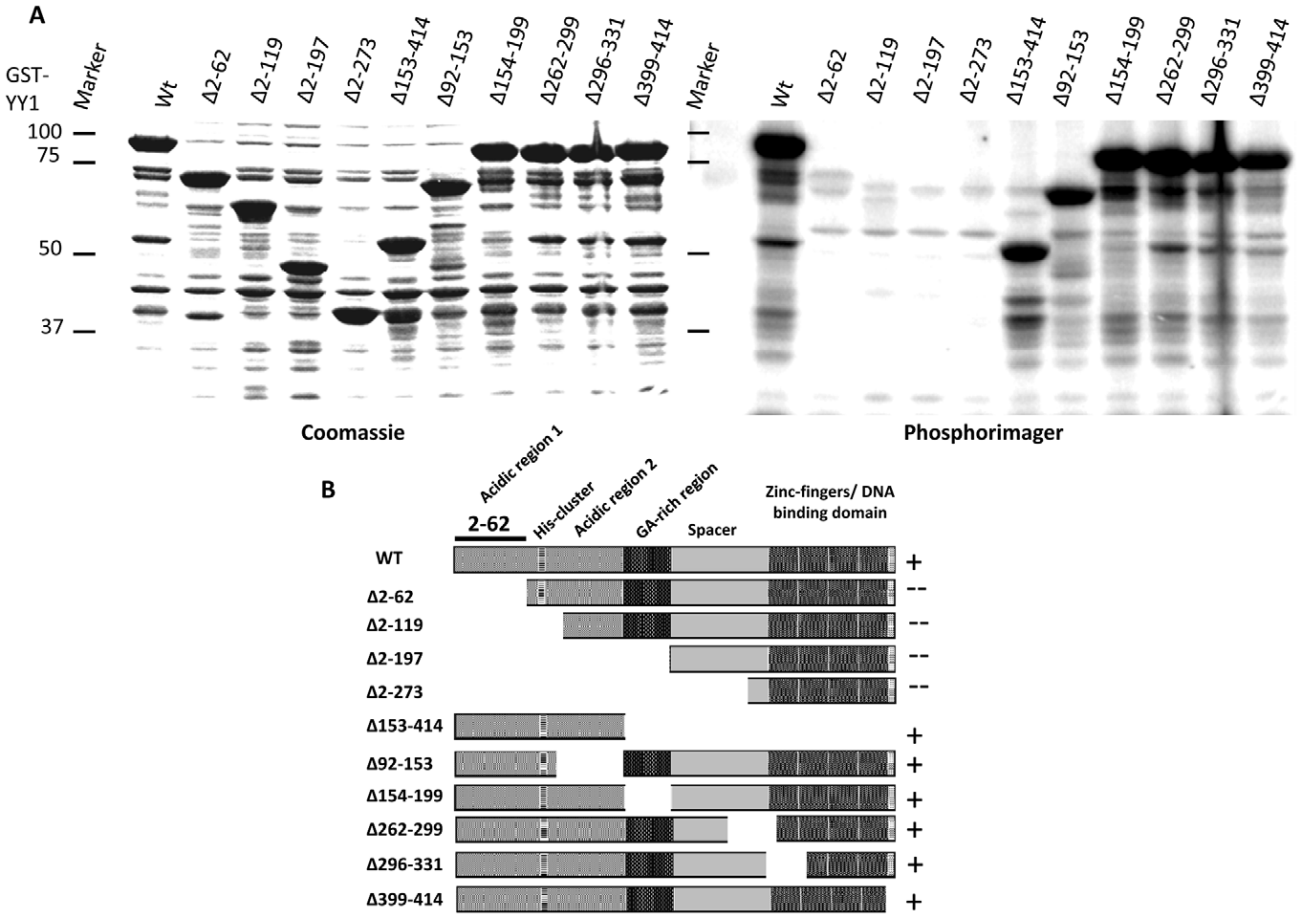
Plk1 phosphorylates YY1 in the N-terminal activation domain *in vitro*. (**A**) Coomassie blue staining (left) and phosphorimager exposure (right) of SDS-PAGE gel analysis of the radioactive *in vitro* kinase assay reactions using purified Plk1 and a panel of GST-tagged YY1 deletion mutants. The specific YY1 deletions are indicated above the lanes. Equal amounts of purified Plk1 were added to all reactions. (**B**) Diagram of the deletion mutants of YY1 used in the kinase assay in (A). Evidence of phosphorylation shown in (A) is indicated by the (+) sign, whereas the absence of evidence of phosphorylation is indicated by a (--) sign. The region identified as the site for phosphorylation by Plk1 is indicated (amino acid residues 2–62).

First, we used a panel of YY1 deletion mutants, which had a Glutathione-S-Transferase (GST) tag. GST-YY1, full length or various deletion mutants, was overexpressed in bacterial cells, and then bacterial lysates were used in a radioactive *in vitro* kinase assay with purified Plk1. As shown in Figure 2A, Plk1 was able to efficiently phosphorylate full-length YY1. However, phosphorylation was abolished with the deletion of the first 62 amino acid residues. Phosphorylation was also abolished in YY1 mutants having additional deletions from the N-terminal side (2–119, 2–197, and 2–273). Deletion of the entire C-terminal domain (153–414), had no effect on the phosphorylation of YY1; neither did deletions of residues 92–153, 154–199, 262–299, 266–331, and 399–414. A diagram displaying these results is presented in Figure 2B. This evidence indicates that the first 62 amino acid residues of YY1 are crucial for the phosphorylation by Plk1.

**Figure 3:**
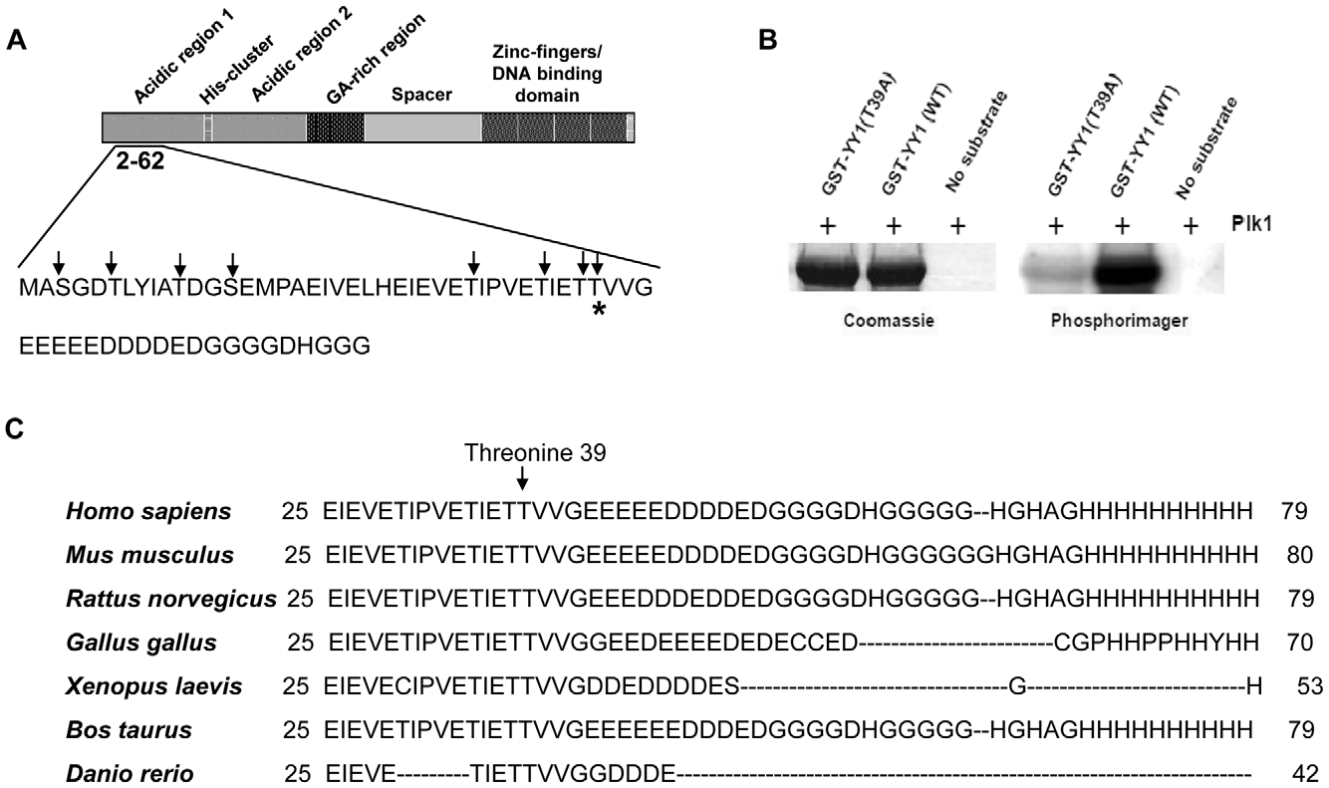
Plk1 phosphorylates YY1 at threonine 39 *in vitro*. (**A**) Diagram displaying the different domains of the YY1 protein. Amino acid residues 2–62 are shown; serine and threonine residues in amino acids 2–62 are indicated by arrows. The predicted phosphorylation site at threonine 39 is indicated with a star. (**B**) Coomassie blue staining and phosphorimager exposure of the SDS-PAGE gel analysis of the radioactive *in vitro* kinase assay reactions. Kinase reactions include Plk1 only (no substrate lane), Plk1 with GST-YY1 wild type (WT) or mutant (T39A). (**C**) Amino acid sequence alignment of the N-terminal domain of the YY1 protein from different species, as indicated.

Then, we analyzed the amino acid composition of the N-terminal residues of YY1. Eight serine and threonine residues were found in this sequence. However, only one threonine, at position 39, resembles a possible Plk1 consensus site (Fig. 3A) [59]. To test if threonine 39 is the Plk1 phosphorylation site, we mutated this residue to alanine, generating a GST-YY1 (T39A) mutant. When tested in a radioactive *in vitro* kinase assay with Plk1, the GST-YY1 (T39A) mutant displayed significantly reduced phosphorylation relative to the wild type (Fig. 3B). Next, we analyzed the conservation of threonine 39 residue of YY1 among different species. We found that it is very well conserved in human, mice, rat, chicken, frog, zebrafish, and cattle (Fig. 3C). In addition, the amino acid residues surrounding this site, and contributing to the formation of a consensus sequence, were also conserved; especially a negatively charged glutamic acid residue at position −2 and an aliphatic residue (valine) at position +1, relative to threonine 39 [59].

### Anti-phospho-T39 antibody specifically recognizes phosphorylation of YY1 by Plk1

**Figure 4:**
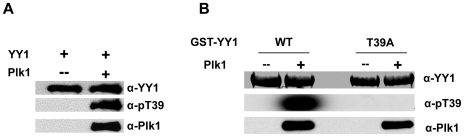
Anti-p-T39 antibody specifically recognizes YY1 phosphorylation at threonine 39. (**A**) Western blot analysis of the cold *in vitro* kinase assay reactions using purified Plk1 and purified YY1, after SDS-PAGE. The blot was probed with anti-YY1, anti-Plk1 and anti-pT39 antibodies. (**B**) Western blot analysis of the cold *in vitro* kinase assay reactions after SDS-PAGE, using purified Plk1 and purified GST-YY1 wild type (WT) or mutant (T39A). The blot was probed with anti-YY1, anti-Plk1 and anti-pT39 antibodies.

To characterize the phosphorylation of YY1 at threonine 39 and study its occurrence in cells, a phospho-specific antibody was raised against a synthetic peptide encompassing YY1 residues 33–44, and carrying a phosphorylation at T39 residue. The polyclonal antibody (anti-pT39) was generated in rabbit, and purified on phosphospecific affinity columns. To test the specificity of anti-pT39 in recognizing the phosphorylation of YY1 by Plk1, we performed a cold *in vitro* kinase assay using purified Plk1 and YY1. The kinase reactions were separated on an SDS-PAGE gel and transferred to a nitrocellulose membrane. When the blot was probed with anti-pT39 antibody, one band was detected in the lane where YY1 was incubated with Plk1 but not in the YY1-only lane (Fig. 4A). Re-probing the blot with YY1 specific antibody confirmed the band as YY1, and showed an equal band in the YY1-only lane. To further test the specificity of the antibody for threonine 39, a second cold kinase assay was performed using GST-YY1 wild type (WT) or mutant (T39A), as substrates. When the reactions were analyzed with Western blotting, and probed with anti-pT39, a band was detected only for GST-YY1 (WT) incubated with Plk1 (Fig. 4B).

### Phosphorylation of YY1 at T39 peaks at G2/M transition

**Figure 5:**
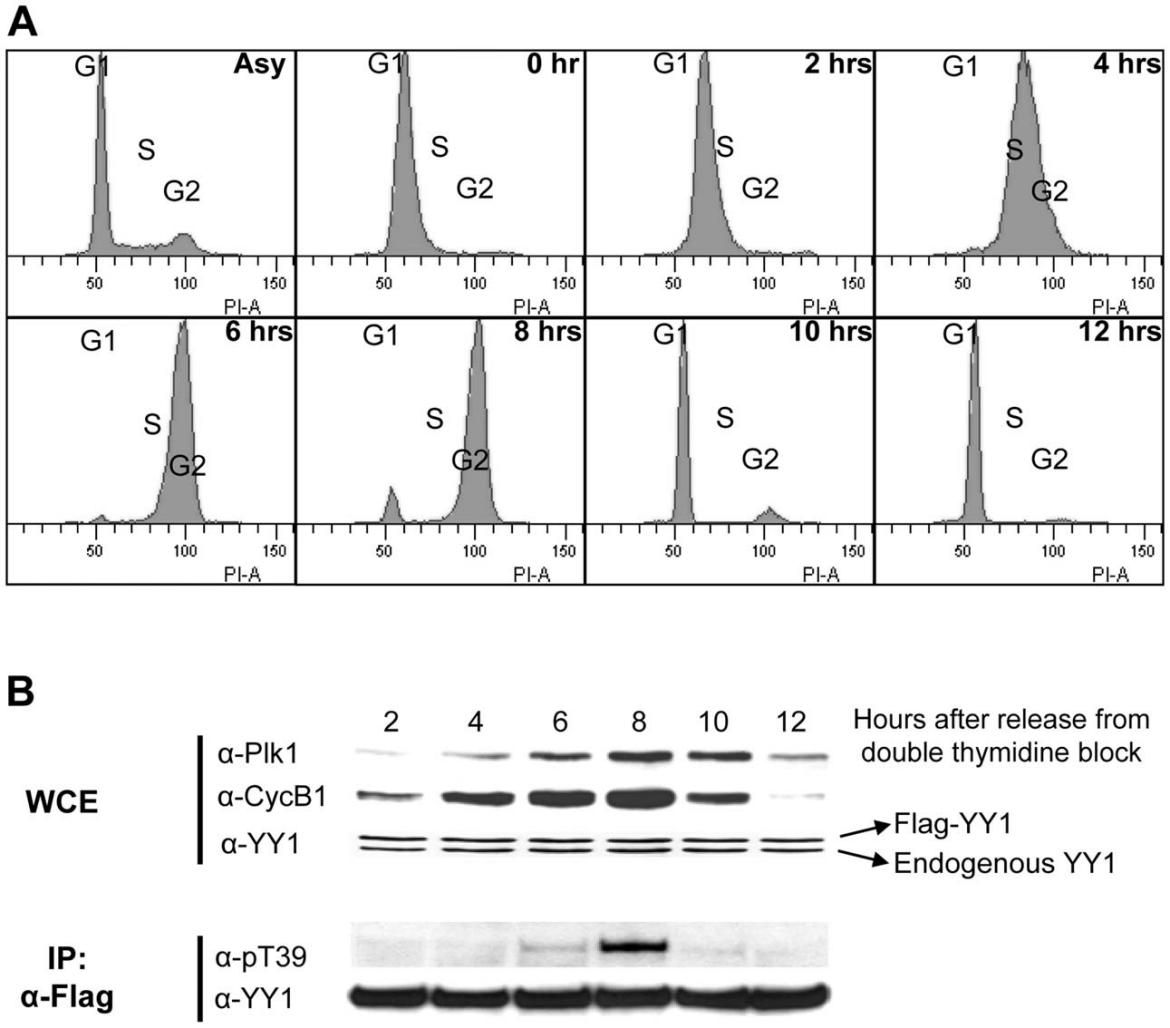
Threonine 39 phosphorylation on YY1 peaks at G2/M transition. Stable HeLa-Flag-YY1 cells were synchronized by double-thymidine block and then released. (**A**) Analysis of the cell-cycle progression of HeLa cells released after double-thymidine block using fluorescence-activated cell sorting. Cells were stained with propidium iodide and analyzed based on their DNA content. An asynchronous population of cells was used as a control. (**B**) Whole cell extracts were prepared from HeLa-Flag-YY1 cells collected at the indicated times after release from double-thymidine block. Total WCE were analyzed on a Western blot after SDS-PAGE separation, and probed with anti-Plk1, anti-Cyclin B1, and anti-YY1 antibodies. Flag-YY1 was immunoprecipitated from the extracts of each time point, and then analyzed on a Western blot using anti-pT39 and anti-YY1 antibodies.

**Figure 6:**
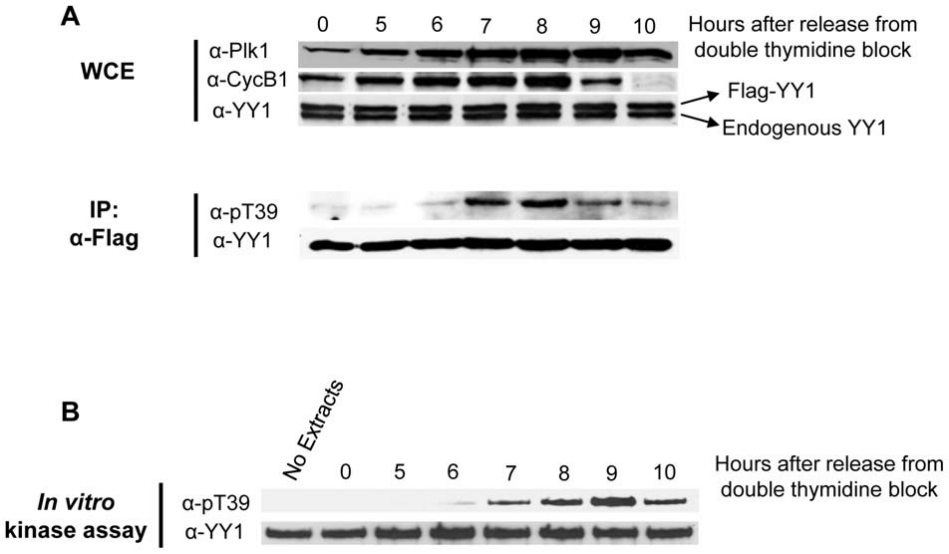
YY1 phosphorylation at T39 is rapidly dephosphorylated upon entry into mitosis. Stable HeLa-Flag-YY1 cells were synchronized by double-thymidine block and then released. Cells were collected for WCE preparation at the indicated time points. (**A**) Western blot analysis of HeLa WCEs and immunoprecipitated Flag-YY1 at the indicated time points. WCEs were probed with anti-Plk1, anti-Cyclin B1, and anti-YY1 antibodies. Immunoprecipitated Flag-YY1 was probed with anti-pT39 and anti-YY1 antibodies. (**B**) *In vitro* kinase assay using the same WCEs tested in (A) at the indicated time points with GST-YY1 attached to beads, in the presence of phosphatase inhibitors. Reactions were separated on SDS-PAGE, transferred to nitrocellulose membrane, and probed with anti-pT39, then anti-YY1 antibody.

The next question is whether this modification occurs on YY1 *in vivo* and if it is cell cycle regulated. For this purpose we synchronized HeLa cells stably transfected with Flag-YY1 [31], using double-thymidine block, as described in the methods section. After the second block, cells were released into fresh medium and samples were collected either for cell cycle analysis using propidium iodide staining followed by Fluorescence Activated Cell Sorting (FACS) analysis, or for whole cell extract (WCE) preparation. Figure 5A shows the cell cycle distribution of cells as they progress from the block. An asynchronous population of HeLa-Flag-YY1 was also analyzed by FACS, as a control. Cells blocked with double-thymidine show early S-phase content of DNA. After release, cells progressed into S-phase, at 2 hours and 4 hours. At 6 hours, cells appear to have G2 level of DNA content, and at 8 hours, they appear to be at late G2 and moving through G2/M into mitosis. At 10 hours, most cells had exited mitosis and entered G1. Twelve hours after release, all cells were in G1 of the new cell cycle (Fig. 5A). An analysis of the synchronized cell extracts on a Western blot showed that the levels of Plk1 and Cyclin B1 increased as cells progress into S-phase and G2, and peaked at G2/M of the cell cycle. Both Plk1 and Cyclin B1 levels decreased as cells exited from mitosis at 12 hours. Decrease in Cyclin B1 levels started at 10 hours, while Plk1 levels remained high until the 12 hour time point. Both endogenous YY1 and Flag-YY1 protein levels remain constant (Fig. 5B). These results are consistent with previous reports by several groups [67], [68], [69]. To analyze the threonine 39 phosphorylation on YY1, Flag-YY1 was immunoprecipitated from WCEs prepared from each time point, loaded on an SDS-PAGE gel, transferred to a nitrocellulose membrane, and probed with anti-pT39 and anti-YY1 antibodies. Although the level of immunoprecipitated YY1 was equal in each time point, detection of T39 phosphorylation was mainly at the 8 hour time point (Fig. 5B), in correlation with the high peak of Plk1 protein levels. This also correlates with previous reports of the timing of the high peak of Plk1 kinase activity [68], [70]. Interestingly, the levels of YY1 T39 phosphorylation decreases sharply as cells progress into mitosis, which shows that this phosphorylation has a very specific temporal occurrence at G2/M. To reproduce this result and further analyze the timing of the T39 phosphorylation, the same experiment was repeated, but cells were collected at time points 5, 6, 7, 8, 9, and 10 hours (Fig. 6). Similar results were obtained for Plk1 and YY1 levels. Cyclin B1 levels were shown to decrease sharply between 8–9 hours, as expected for a well synchronized population of cells, and it marks the transition through prophase, metaphase and into anaphase [69]. Following this same time line, T39 phosphorylation of YY1 also decreased sharply between 8 to 9 hours (Fig. 6A). This is particularly interesting, because the levels of Plk1 remain high at these time points. However, it has been reported previously that Plk1 changes its cellular localization and substrate specificity at later stages of mitosis, playing specific roles for the proper execution of cytokinesis [40], [66]. To test if Plk1 at the 9 and 10 hour-time points is active and can phosphorylate YY1, we performed an *in vitro* kinase assay using the same timed cell extracts in panel A with GST-YY1 as a substrate. As shown in Figure 6B, GST-YY1 was efficiently phosphorylated by the 9 hour-time point extracts, and to a lesser extent by the 10 hour-time point extracts.

**Figure 7:**
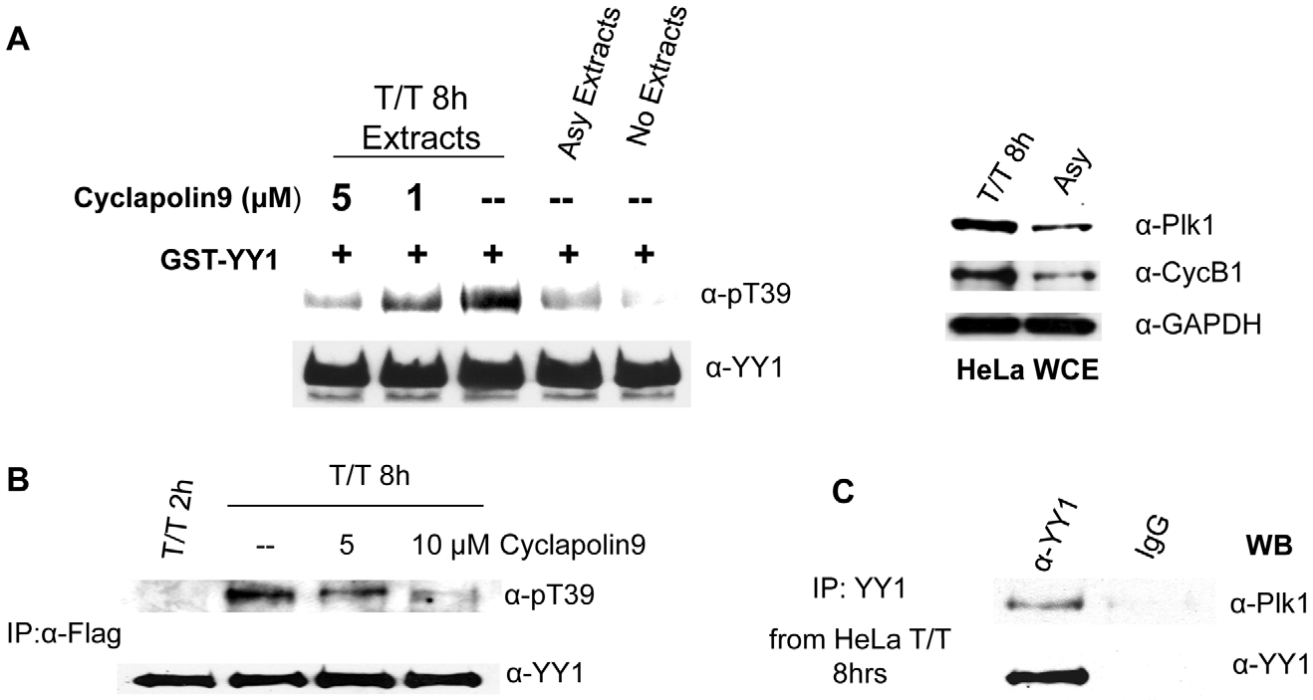
Plk1 phosphorylates YY1 at threonine 39 *in vivo*. (**A**) Western blot analysis of cold *in vitro* kinase assay reactions using HeLa whole cell extracts (WCE) as the source for kinase activity and bacterially expressed GST-YY1 bound to glutathione beads, as substrate. WCEs were prepared from HeLa cells, asynchronously growing or double-thymidine blocked and released for eight hours (T/T 8 h). Plk1 inhibitor, Cyclapolin 9, was added to the kinase reactions of T/T 8 h extracts at the indicated concentrations. Reactions were separated on SDS-PAGE, transferred to nitrocellulose membrane, and probed with anti-pT39, then anti-YY1 antibody. WCEs were also analyzed on a separate Western blot (right panel), using anti-Plk1 and anti-Cyclin B1 antibodies. Anti-GAPDH was used as a loading control. (**B**) Flag-YY1 was immunoprecipitated from HeLa-Flag-YY1 cells, synchronized by double-thymidine block and released for eight hours. Cyclapolin 9 (or DMSO, for the negative control for the inhibitor) was added to the cells four hours prior to cell collection. Flag-YY1 was also immunoprecipitated from cell extracts collected two hours after release as a negative control. The resulting Western blot was probed with anti-pT39 and anti-YY1 antibodies. (**C**) Co-immunoprecipitation of Plk1 with YY1 from WCEs prepared from HeLa cells released for eight hours after double thymidine block. YY1 was immunoprecipitated using an antibody specific for the last 20 amino acids of the YY1 (C-20). IgG was used as a control for the specificity of the immunoprecipitation. The Western blot was probed with anti-Plk1 and then anti-YY1 antibodies.

Next, we wanted to provide more *in vivo* evidence that YY1 is a substrate for Plk1, specifically at the G2/M transition of the cell cycle. For this purpose we prepared WCEs from HeLa cells, asynchronously growing or double-thymidine blocked and released for 8 hours (T/T 8 h). Synchrony at G2/M transition was verified using FACS analysis (data not shown). In addition, Western blot analysis showed higher levels of Plk1 and Cyclin B1 in T/T8 h extracts, relative to asynchronous extracts (Fig. 7A, right panel). Then, asynchronous or T/T8 h extracts were used in a cold *in vitro* kinase assay with GST-YY1, in the absence or presence of Cyclapolin 9, a potent and highly specific Plk1 inhibitor. The kinase reactions were analyzed by Western blot, using anti-pT39 and anti-YY1 antibodies. Figure 7A shows that incubation of GST-YY1 with the WCEs from T/T8 h results in much higher T39 phosphorylation than the WCEs from the asynchronous population, correlating with the high level of Plk1 activity. Moreover, the addition of Plk1 inhibitor (Cyclapolin 9) [71] greatly reduced GST-YY1 T39 phosphorylation; indicating that the corresponding kinase activity in T/T8 h extracts was indeed due to Plk1. Next, we synchronized HeLa-Flag-YY1 cells with double-thymidine, released, and harvested at 8 hours after release, with or without the addition of Cyclapolin 9 at 4 hours after release. Two different concentrations of Cyclapolin 9 were added, 5 and 10 µM. WCEs were prepared, and Flag-YY1 was immunoprecipitated and analyzed on a Western blot with anti-pT39 and anti-YY1 antibodies. The levels of immunoprecipitated Flag-YY1 were shown to be equal; however, the level of T39 phosphorylation sharply decreased upon addition of Plk1 inhibitor (Fig. 7B). Flag-YY1 was also immunoprecipitated from WCE of cells collected at 2 hours after release as a negative control for the phosphorylation (T/T 2 h). To further prove that YY1 is an *in vivo* substrate of Plk1, we performed co-immunoprecipitation experiments using WCEs from HeLa cells which were double-thymidine blocked and released for 8 hours. We observed that Plk1 co-immunoprecipitated with endogenous YY1, using a C-terminal YY1 specific antibody (Fig. 7C). This is direct evidence for an *in vivo* physical interaction between YY1 and Plk1 at G2/M transition of the cell cycle.

## Discussion

Strict control of entry into mitosis is essential for the proper execution of cell division and the accurate distribution of the genetic material to the two daughter cells. This control is ordered by an elaborate network of phosphorylation signaling pathways, and positive and negative feedback loops. Disruption of these pathways can lead to fatal diseases in humans, such as cancer. A better understanding of these pathways and their regulation can lead to enhanced therapies.

The transcription factor YY1 has been shown to be one of the major regulators in development and cell proliferation. An abundance of evidence in the literature supports the critical roles of YY1 in the transitions through the different phases of the cell cycle [1], [2], [63]. However, the mechanisms for the modulation of these roles are not clear. Expression of the *yy1* gene and YY1 protein levels do not fluctuate throughout the cell cycle. Most hypotheses proposed for the different roles and temporal regulation of YY1 have pointed to external elements, like interactions with proteins that in turn are cell cycle regulated. Our research has focused on posttranslational modifications which can occur on YY1 in a cell cycle dependent manner. For this purpose, we analyzed the amino acid sequence of YY1 and searched for consensus sites for phosphorylation by different cell cycle kinases. One of the kinases that showed high probability for phosphorylation of YY1 was Plk1. This prediction seemed reasonable due to the functional overlap between YY1 and Plk1 in the proper progression of mitosis and cytokinesis [16].

To test this prediction, we performed a series of radioactive *in vitro* kinase assays. We showed that YY1 is a good substrate for Plk1 and we mapped the phosphorylation site to theonine 39 of YY1. Then, we developed a phospho-specific antibody that can recognize phosphorylated threonine 39 (anti-pT39). This antibody showed high specificity for phospho-T39 on YY1, when phosphorylated by Plk1 *in vitro*.

When whole cell extracts from asynchronous HeLa cells were tested on Western blot, the antibody produced several cross-reacting bands (data not shown). Therefore, we tested this antibody on immunoprecipitated Flag-tagged YY1, from different time points of the cell cycle. Anti-pT39 recognized phosphorylated Flag-YY1 only in late G2, and mainly at G2/M transition, although the levels of immunoprecipitated YY1 were identical at all time points (Fig. 5). The inability of the antibody to recognize phosphorylation on YY1 in asynchronous WCE is likely due to the low stoichiometry of this phosphorylation, since it occurs during a very short time interval of the cell cycle. Also, it possibly occurs only on a limited subpopulation of YY1 protein.

Many Plk1 substrates have been shown to be primed for phosphorylation by Plk1 by other kinases [58], [66]. Since Plk1 strongly phosphorylated bacterially expressed YY1 *in vitro*, it does not appear to require a priming phosphorylation. However, this does not exclude the possibility that priming of YY1 on a different site *in vivo* could play an important role in modulating this phosphorylation.

An interesting finding in this study was the rapid dephosphorylation of threonine 39, as cells progress into mitosis. In contrast to this rapid dephosphorylation, the levels of Plk1 remain high well beyond the time of the loss of T39 phosphorylation, as shown in our assays. Plk1 activity has been known to persist throughout mitosis and cytokinesis [41], [67], [68], [70]. This is also supported by our *in vitro* kinase assay, in which Plk1 in cell extracts from the 9 hour time point can efficiently phosphorylate YY1 *in vitro*. Therefore, it is possible that a strong phosphatase activity somehow overcomes the phosphorylation of YY1 by Plk1, and that Plk1 only phosphorylates YY1 at the entry into mitosis *in vivo*. These possibilities are not mutually exclusive, but rather more probably coordinated. The cellular distribution and changes of localization of Plk1 at later stages of mitosis have been shown to play a significant role in its substrate recognition [40], [41], [58], [66]. This indicates that Plk1 phosphorylation of YY1 has a very precise role at this point of the cell cycle, and it could be an indirect mechanism through which Plk1 regulates the expression of proteins needed later in cytokinesis. Evidence that YY1 is a physiological substrate for Plk1 is strengthened by the physical interaction between YY1 and Plk1 shown by co-immunoprecipitation. Also, selective inhibition of Plk1 phosphorylation of YY1 by Cyclapolin 9 [71] is consistent with the conclusion that Plk1 is the kinase responsible for T39 phosphorylation.

The timing of threonine 39 phosphorylation functionally overlaps with YY1 regulation of genes activated at G2/M. Overexpression of Flag-YY1, where threonine 39 is mutated to alanine (non-phosphorylatable) or to aspartic acid (phosphomimicking), did not produce significant phenotypic effects in HeLa cells (data not shown). This could be due to the abundance of endogenous wild type YY1 or the need for other modifications on YY1 to produce phenotypic effects. Future studies will help reveal the exact function of this modification.

In summary, we reveal in this report the first identification of a kinase which phosphorylates YY1. This phosphorylation occurs in the activation domain of YY1 and is cell cycle regulated. We show, *in vitro* and *in vivo*, that YY1 is a good substrate for Plk1 and implicate YY1 in the cascade of signaling events that take place at G2/M transition. A better understanding of all the elements involved in the entry into mitosis is of critical importance in enhancing targeted cancer therapies.

## Materials and Methods

### Cell Culture

HeLa S3 and HeLa-Flag-YY1 [31] cells were grown at 37°C in 5% CO_2_ in Dulbecco's Modified Eagle Medium (DMEM) (Cellgro, Herndon, VA) supplemented with 10% Fetal Bovine Serum (FBS) (FBS; Mediatech, Herndon, VA), 1% Non-Essential amino acids (Sigma, St. Louis, MO) and 1% Penicillin-Streptomycin (Mediatech). HeLa-Flag-YY1 cells are stably transfected with pCS2(+)-Flag-YY1. Double-thymidine synchronization and establishment of HeLa-Flag-YY1 stable cell line was described previously [31].

Whole Cell Extract (WCE) preparation, Immunoprecipitation (IP), Western blotting and Electrophoretic-Mobility Shift Assays (EMSA) were performed as previously described [31], [63]. Antibody used for IP was anti-YY1(C-20); antibodies used for Western blotting were anti-YY1 (H-10), anti-Plk1, anti-Cyclin B1, anti-GAPDH (Santa Cruz Biotechnology, Santa Cruz, CA). The rabbit polyclonal anti-pT39 was generated by New England Peptide using a synthesized phospho-peptide corresponding to amino acids 33–44 of YY1 (Ac-VETIET(pT)VVGEEC-amide).

### Plasmid construction

#### pGEX-2T-YY1

For the construction of GST-tagged YY1 bacterial expression plasmid, an NcoI/EcoI fragment encompassing the open reading frame of human YY1 was moved from pCMV-HA-YY1 (a gift from Dr. Bernhard Lüscher, Aachen University, Germany) [5] by digestion, gel purified, and inserted into a BamHI/EcoRI digest of pGEX-2T vector (Amersham Pharmacia), after blunting the NcoI and BamHI sites of the insert and vector, respectively. The NcoI and BamHI sites were blunted using the Klenow fragment of Pol I (NEB).

#### pET-20b-YY1

For the construction of the YY1 bacterial expression plasmid, the NcoI/EcoRI fragment of pGEX-2T-YY1, described above, was subcloned into the multiple-cloning region of pET-20b+ vector (Invitrogen, Carlsbad, CA). A sequence encoding pelB leader secretion peptide is found upstream of the multiple-cloning region. To remove this sequence, pET-20b+ was digested with NdeI/NcoI, then blunted and religated. This construct, once expressed in bacterial cells would produce non-tagged YY1 protein.

### Bacterial expression and purification of YY1

#### GST-YY1

Rosetta (DE3) cells (Novagen) were transformed with the pGEX-2T-YY1 construct and grown overnight in LB Miller broth medium (EMD) with ampicillin (100 µg/ml final concentration). The overnight culture was diluted 1∶10 in the same medium (with ampicillin) and grown to a density of 0.6 O.D. (about 1 hour), then induced with isopropyl β-D-1-thiogalactopyranoside (IPTG-Sigma-Aldrich) at a final concentration of 0.5 mM for about 4 hours. Cells were pelleted by centrifugation and then resuspended in lysis buffer (ice-cold phosphate-buffered saline (PBS) pH 8.0 or 50 mM Tris pH 8.0, 150 mM NaCl) supplemented with a cocktail of protease inhibitors (Sigma). The suspension was sonicated on ice (three bursts, 15 seconds each, with 2 minutes intervals between sonication bursts to allow cooling). Lysates were cleared by centrifugation, then incubated with immobilized glutathione beads (Pierce) with rocking for 2–4 hours at 4°C. The resulting slurry of beads and lysates was centrifuged at 500×g for 2 minutes at 4°C. The beads were washed 3 times with lysis buffer. GST-YY1 was then eluted at 4°C from the beads with an equal volume of 50 mM Tris pH 8.0 containing 15 mM reduced glutathione (Sigma). GST-YY1 protein was aliquoted and stored at −80°C until used.

#### YY1

Expression conditions for the pET-20b-YY1 plasmid were identical to those described above for the pGEX-2T-YY1 plasmid. However, after pelleting the Rosetta cells, the cells were resuspended in denaturing buffer (8 M Urea, 100 mM NaH_2_PO_4_, 10 mM Tris, pH 8.0), and allowed to lyse, rocking for 45 minutes at room temperature (RT). Lysates were cleared by centrifugation at 18000×g, at RT for 30 minutes. Nickel-nitrilotriacetic (Ni-NTA) beads (Pierce), prewashed 3 times with 10 volumes each time with lysis buffer, were added to the cleared lysates and rocked for 1 hour at RT. The slurry of beads and lysates was then poured into a column, and the beads were allowed to settle. Lysates were drained by gravity. Ni-NTA beads were then washed one time with 10 volumes of lysis buffer pH 8.0, followed by 3 washes with lysis buffer pH 6.3. YY1 protein was eluted from the column with lysis buffer pH 4.5, after first lowering the pH to 5.9. After analysis of samples from the elution fractions on a 10% SDS-PAGE gel, by Coomassie blue staining, YY1-containing fractions were pooled and dialyzed to renature the protein. Renaturation was performed by dialysis against 50 mM Tris pH 7.4, 150 mM NaCl, 1 mM ZnSO_4_, 5 mM β-Mercaptoethanol, 1 M urea, for 2 hours. Another dialysis step followed using the same buffer except that the urea concentration was lowered to 0.1 M. Two additional dialysis steps were performed in buffer lacking urea and β-mercaptoethanol, for 2 hours each time. Dialysis in this solution was repeated one more time overnight at 4°C. The renatured YY1 protein was aliquoted and stored at −80°C for later use.

### Mutagenesis

Generation of YY1 point mutations at threonine 39 to alanine was performed using pET-20b(+)-YY1 plasmid and then subcloned into pGEX-2T-YY1. Mutagenesis was performed using QuikChange II Site-Directed Mutagenesis Kit (Stratagene, La Jolla, CA), according to the manufacturer's instructions. The threonine to alanine mutation was confirmed by sequencing. The primers used for the mutagenesis of threonine 39 to alanine substitution were:

Sense:

5′-GACCATCGAGACC**G**CAGTGGTGGGCGA-3′

Antisense:

5′-TCGCCCACCACTG**C**GGTCTCGATGGTC-3′

### *In vitro* kinase assays

Kinase reactions were performed in kinase buffer (50 mM Tris pH 7.4, 10 mM MgCl_2_, 50 µM ATP, 0.25 µM ^32^P-γ-ATP, 5 mM beta-glycerophosphate, 10 mM NaF, 1 mM DTT) for 30 minutes at 30°C, with shaking. Purified Polo-like kinase 1 (Plk1) was purchased from SignalChem (British Columbia, Canada). Reactions were then stopped by the addition of SDS-PAGE buffer and loaded for separation on a 10% SDS-PAGE gel. After staining with Coomassie Brilliant Blue R-250, to visualize the protein bands, gels were dried and exposed overnight to a Phosphorimager screen at room temperature. The screen was then scanned on a Typhoon 9410 imager (GE Healthcare, Waukesha, WI) for analysis.

For the cold kinase assays, no radioactive ATP was added, and the cold ATP concentration was raised to 2 mM. After separation on the SDS-PAGE gel, proteins were transferred to a nitrocellulose membrane and probed with the indicated antibodies.

For the kinase assays using HeLa whole cell extracts as the kinase source, extracts were added to GST-YY1 attached to glutathione beads in kinase buffer. After incubation, the beads-GST-YY1 complexes were pelleted by centrifugation, and cell extracts we aspirated. Beads were washed 2× with kinase buffer, and then boiled in 2× SDS-PAGE loading buffer, prior to loading on the gel and subsequent Western blotting.
